# Cardiopulmonary Exercise Testing in the Differential Diagnosis Between Athlete’s Heart and Cardiac Pathology: Current Evidence and Diagnostic Challenges

**DOI:** 10.3390/diagnostics16162573

**Published:** 2026-08-14

**Authors:** Bogdan Caloian, Carmen Silvia Caloian, Raluca Tomoaia, Diana Andrada Irimie, Florina Iulia Fringu, Dan Horatiu Comsa, Gabriel Laurentiu Cismaru, Gabriel Nicolae Gusetu, Radu Ovidiu Rosu, Dana Pop

**Affiliations:** 1 Department of Cardiology-Rehabilitation, Faculty of Medicine, University of Medicine and Pharmacy “Iuliu Hatieganu”, Cluj-Napoca 400347, Romaniaflorina.fringu@yahoo.com (F.I.F.); dh.comsa@gmail.com (D.H.C.); gabi_cismaru@yahoo.com (G.L.C.); gusetu@gmail.com (G.N.G.); rosuradu1053@gmail.com (R.O.R.);; 2 Department of Periodontology, Faculty of Dental Medicine, University of Medicine and Pharmacy “Iuliu Hatieganu”, Cluj-Napoca 400347, Romania

**Keywords:** hypertrophic cardiomyopathy, dilated cardiomyopathy, arrhythmogenic cardiomyopathy, pectus excavatum, oxygen pulse, ventilatory efficiency, sports cardiology, sudden cardiac death

## Abstract

Sports cardiologists frequently face the challenge of distinguishing physiological cardiac adaptation to intensive training (“athlete’s heart”) from early or mild cardiac pathology, a dilemma in which both false-positive and false-negative assessments carry meaningful consequences for the athlete. Cardiopulmonary exercise testing (CPET) provides a dynamic, functional complement to structural and electrical assessment, but its value in this specific differential-diagnosis context has not been comprehensively synthesized. This narrative review examines current evidence on CPET in distinguishing athlete’s heart from hypertrophic cardiomyopathy, dilated cardiomyopathy, arrhythmogenic cardiomyopathy, and thoracic wall deformities such as pectus excavatum, alongside the physiological basis of high exercise capacity in athletes and the athlete-specific reference values now becoming available. Across conditions, peak oxygen uptake alone proved an unreliable discriminator. Ventilatory efficiency, oxygen-pulse kinetics, and the exercise arrhythmic and blood-pressure response appear to carry additional information when interpreted within a multiparametric framework, although the supporting studies are observational, mostly single-centre, and were not designed to compare these variables against one another. Special populations, including veteran athletes, women, and those with congenital heart disease, require dedicated reference data that remain incomplete, and emerging artificial-intelligence-based interpretive tools require athlete-specific validation before clinical adoption. A practical, stepwise diagnostic framework integrating CPET with imaging and shared decision-making is proposed. It reflects the authors’ synthesis of the reviewed evidence rather than a prospectively validated or society-endorsed pathway. Closing the evidence gaps identified here, particularly for underrepresented athlete populations and prospective outcomes data, should be a priority for future research in sports cardiology.

## 1. Introduction

Regular physical activity and structured athletic training confer well-established cardiovascular, metabolic, and psychological benefits, and participation in organized sport continues to expand across all age groups and competitive levels. Sustained high-volume training induces a spectrum of structural and functional cardiac adaptations collectively termed “athlete’s heart,” including chamber enlargement, mild-to-moderate increases in wall thickness, and electrical remodelling that can be reflected on the resting electrocardiogram (ECG) [1,2]. These adaptations are generally benign and reversible with detraining. In a subset of individuals, however, they overlap phenotypically with early or mild forms of inherited cardiomyopathies, channelopathies, and other structural heart disease that predispose to exercise-related sudden cardiac death (SCD) [3].

This overlap places sports cardiologists in a recurring diagnostic dilemma. A false-negative assessment, in which underlying pathology goes unrecognized, may expose an athlete to a preventable risk of SCD during training or competition. A false-positive assessment, in which a physiological adaptation is misclassified as disease, can instead lead to unwarranted disqualification from sport, unnecessary further testing, psychological distress, and loss of athletic career opportunities [3,4]. Both outcomes carry meaningful consequences, and neither can be avoided simply by adjusting the overall level of diagnostic caution.

Contemporary pre-participation cardiovascular screening protocols typically combine a structured history and physical examination with a resting 12-lead ECG, interpreted according to standardized, athlete-specific criteria that separate normal training-related findings from findings that warrant further investigation [2,4]. When findings are borderline or symptoms are present, morphological imaging with echocardiography and, increasingly, cardiac magnetic resonance is used to characterize chamber size, wall thickness, and tissue characteristics [4]. Structural imaging alone, however, often cannot fully resolve the diagnostic uncertainty in borderline cases, because the morphological “grey zone” between extreme physiological remodelling and mild pathology can be genuinely difficult to separate on anatomical grounds, even with long-term serial follow-up [5].

**Figure 1:**
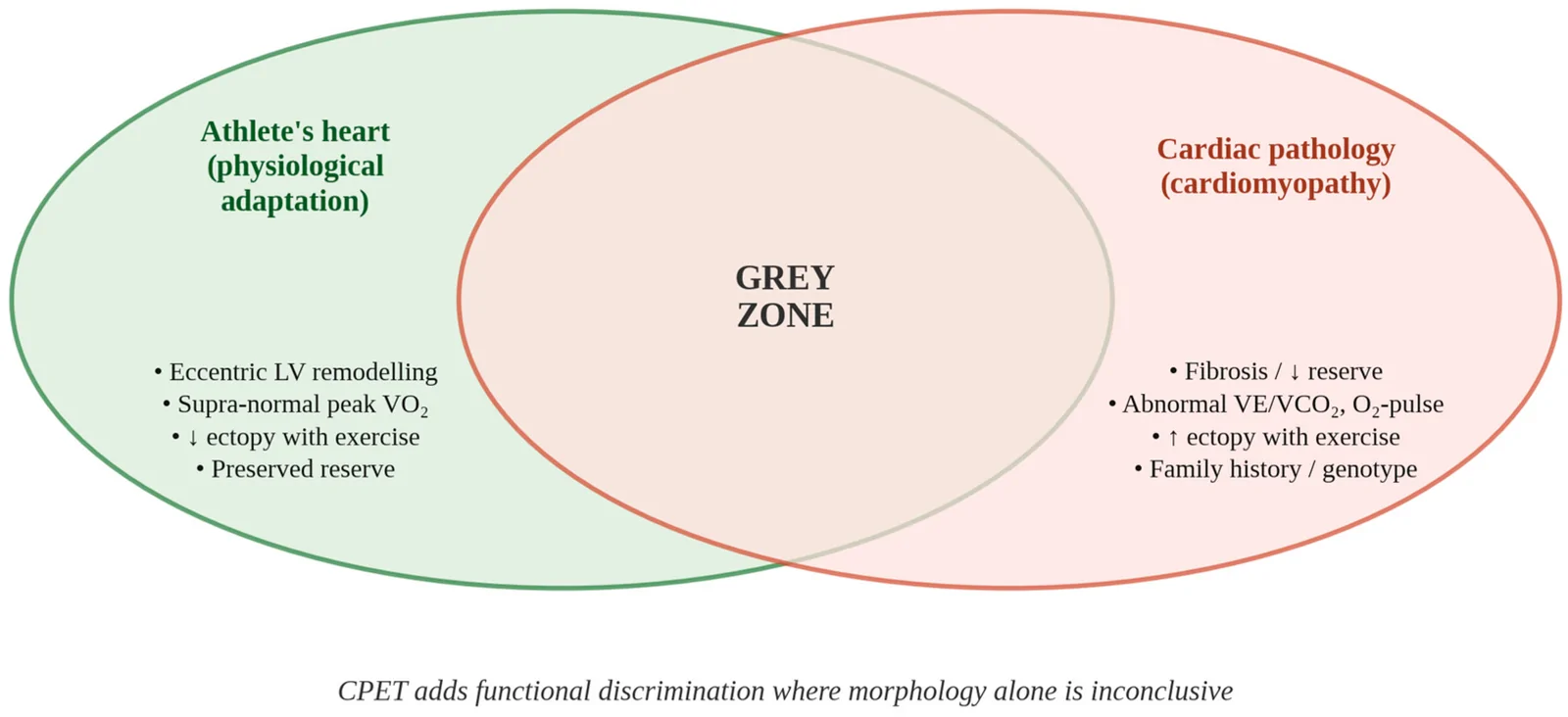
Conceptual overlap between physiological cardiac adaptation and cardiac pathology in athletes. Features listed under each entity are characteristic rather than absolute; substantial overlap defines the diagnostic “grey zone,” where cardiopulmonary exercise testing adds functional information beyond resting morphology. LV, left ventricular; VO_2_, oxygen uptake; VE/VCO_2_, ventilatory equivalent for carbon dioxide.

Cardiopulmonary exercise testing (CPET) offers a complementary, functional dimension to this assessment. By combining a graded exercise protocol with breath-by-breath analysis of oxygen uptake, carbon dioxide output, and ventilation, alongside continuous ECG and blood pressure monitoring, CPET provides an integrated, dynamic readout of cardiac, pulmonary, and skeletal muscle performance under physiological stress that static imaging cannot replicate [6]. Because it interrogates the same physiological reserve that is engaged during genuine athletic effort, CPET can, in principle, add discriminative value where morphological criteria reach their limits. As this review will discuss, however, that potential is not yet matched by a fully standardized, athlete-specific evidence base. The conceptual overlap that CPET is asked to resolve is summarized in Figure 1.

Several gaps currently limit the routine use of CPET as a differential-diagnosis tool in sports cardiology. Reference data are derived predominantly from young, male, endurance-trained cohorts, and other groups, including women, adolescents, veteran athletes, athletes with disabilities, and individuals from diverse ethnic backgrounds, remain comparatively underrepresented. Prospective longitudinal data on athletes with confirmed cardiovascular conditions, which would inform evidence-based eligibility decisions, are scarce. Interpretation criteria for individual CPET parameters are not fully standardized across laboratories, and emerging tools such as artificial-intelligence-assisted threshold detection have not yet been validated in athletic populations.

This narrative review examines current evidence on the use of CPET to distinguish physiological cardiac adaptation from cardiac pathology in athletes. We first summarize the physiological basis of CPET and the reference values available in athletic populations. We then review the diagnostic value of specific CPET parameters in the differential diagnosis of the principal conditions that mimic athlete’s heart, namely hypertrophic cardiomyopathy, dilated cardiomyopathy, arrhythmogenic cardiomyopathy, and thoracic wall deformities such as pectus excavatum, before addressing special populations, emerging technologies, and the evidence gaps that should guide future research. On that basis we also set out a stepwise diagnostic framework, which reflects our own synthesis of the literature rather than a validated or society-endorsed pathway.

## 2. Methods

### 2.1. Literature Search and Study Selection

We searched PubMed, Scopus, and Web of Science for English-language articles published up to January 2026, combining terms related to cardiopulmonary exercise testing (“CPET,” “VO_2_,” “oxygen pulse,” “VE/VCO_2_”) with terms for athlete’s heart and the relevant cardiac conditions (“hypertrophic cardiomyopathy,” “dilated cardiomyopathy,” “arrhythmogenic cardiomyopathy,” “pectus excavatum,” “athlete”). Reference lists of key articles and current guidelines were screened for additional sources.

A narrative review has no protocol to fall back on, so it is worth setting out how we decided what to include. Titles and abstracts were screened by two authors (B.C. and R.T.), and the full text of anything that appeared relevant was read by at least two of us. Disagreements were resolved by discussion within the author group rather than by any formal adjudication rule. We considered original studies, meta-analyses, consensus documents, and society guidelines. Case reports were used sparingly, and only where they illustrated a mechanism that larger studies had not addressed.

Relevance alone did not determine which papers carried weight in the discussion. Where several studies addressed the same question, we gave preference, broadly in this order, to work that recruited athletes rather than general cardiology populations; to larger or multicentre cohorts over single-centre series; to prospective over retrospective designs; and to more recent studies where testing technology or diagnostic criteria had changed materially since the earlier work. Sample size mattered to us less than whether the comparison group was appropriate. A small study with well-matched trained controls was treated as more informative than a larger one that compared athletes with sedentary individuals, since the latter design cannot separate the effect of training from the effect of disease. Reports that gave CPET values without describing the exercise protocol, or without stating whether a maximal effort had been achieved, were read but were not used to support any quantitative statement.

We did not apply a formal quality-appraisal instrument, and we make no claim that the resulting selection is exhaustive. When studies disagreed, our practice was to present both findings and to identify the population each came from, rather than to reconcile them or to adopt the more convincing result. The question of whether patients with dilated cardiomyopathy can meaningfully augment ejection fraction during exercise, where the athlete-derived and veteran-athlete literature point in somewhat different directions, is handled in that way in Section 4.2 [7,8]. Where a single study underpins a statement, we have tried to make that visible in the text. The limitations of this approach are considered further in Section 8.

### 2.2. Use of Artificial Intelligence in Manuscript Preparation

**Figure 2:**
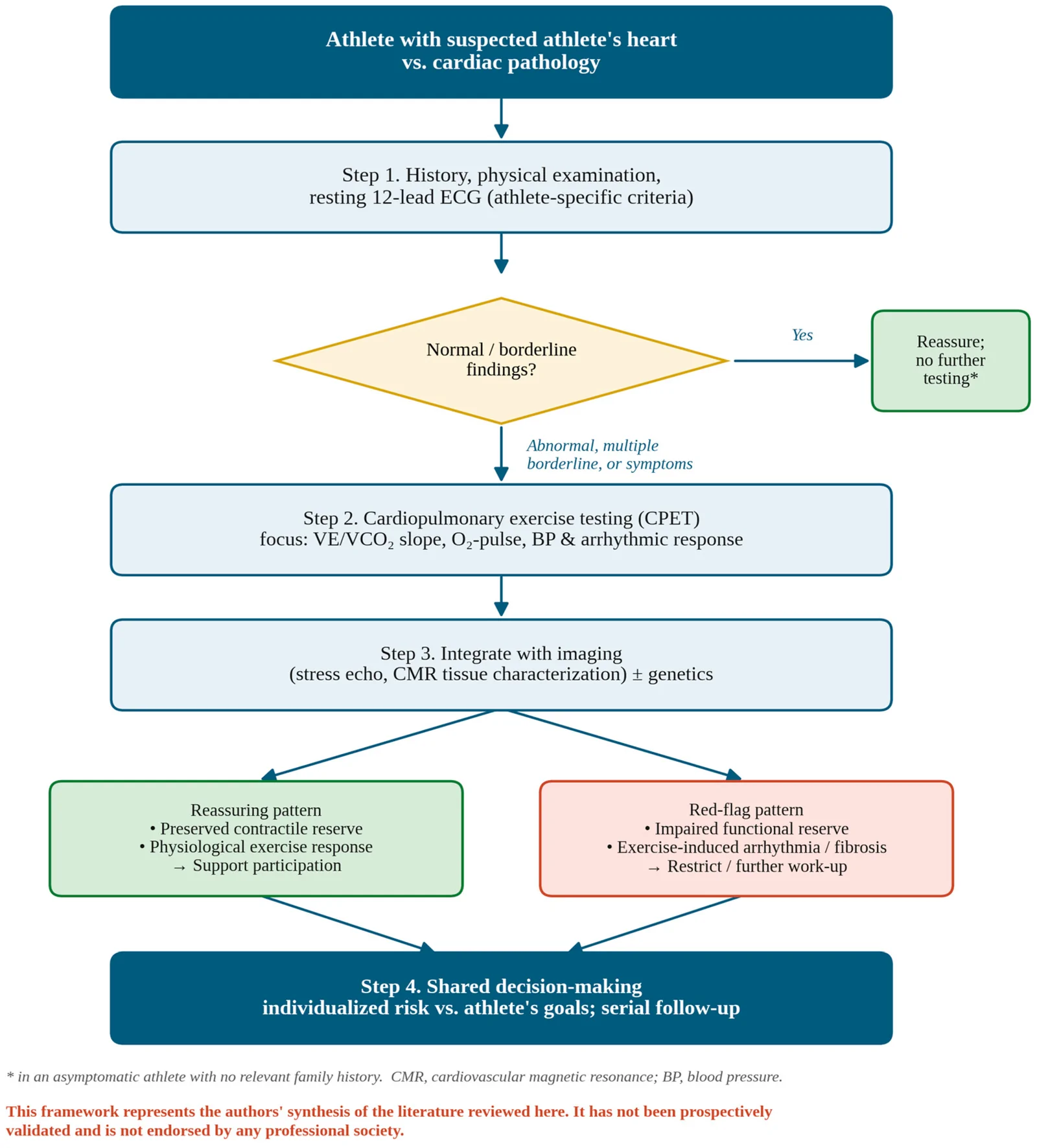
Proposed stepwise framework for integrating cardiopulmonary exercise testing (CPET) into the evaluation of an athlete with suspected overlap between physiological adaptation and cardiac pathology. The figure summarizes the authors’ synthesis of the literature reviewed in this article. It is not a validated algorithm and has not been endorsed by any professional society; the sequence and the relative weight given to each variable represent the authors’ interpretation. CMR, cardiovascular magnetic resonance; BP, blood pressure; VE/VCO_2_, ventilatory equivalent for carbon dioxide.

**Figure 3:**
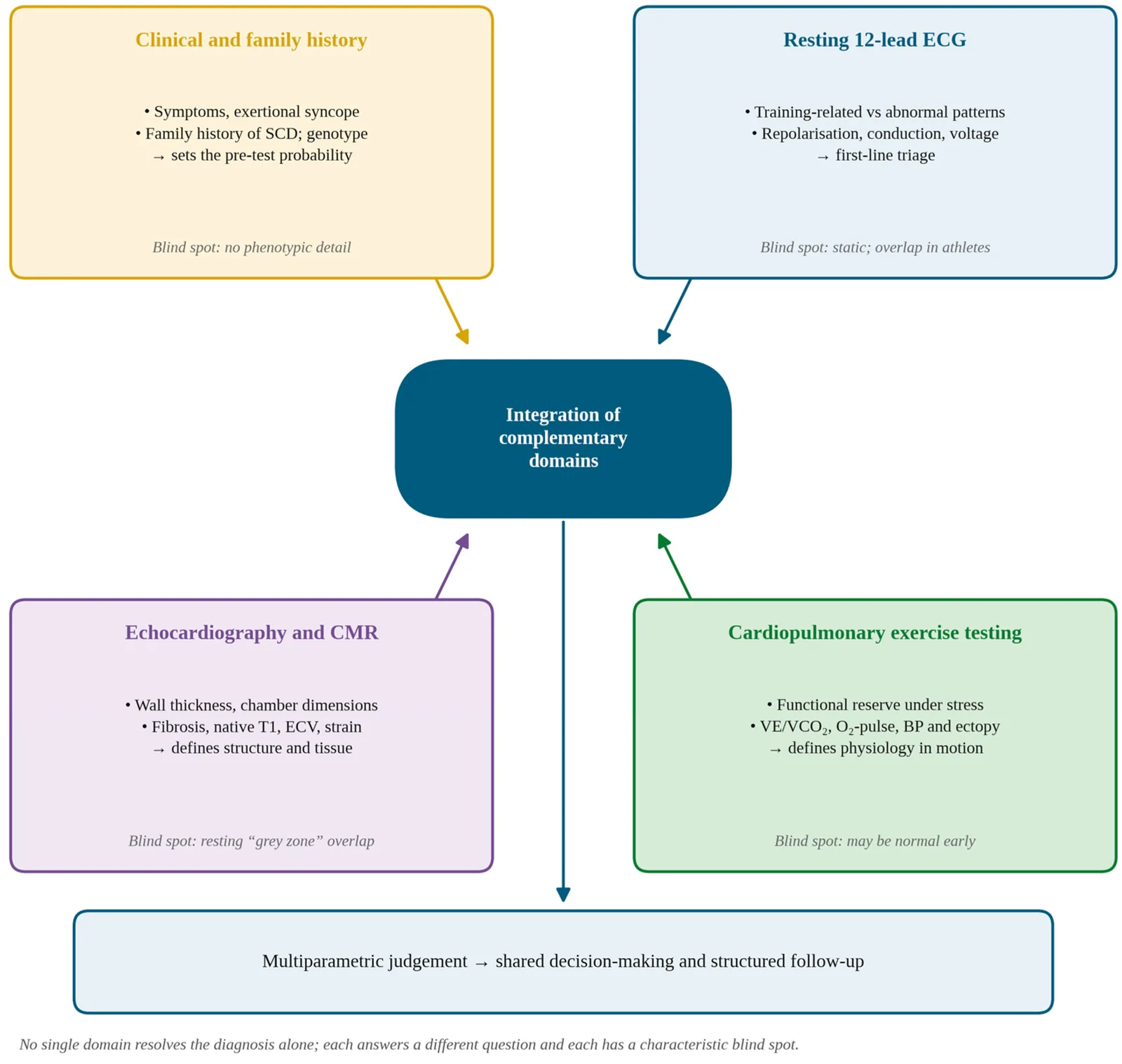
Complementary roles of clinical and family history, the resting electrocardiogram, cardiac imaging, and cardiopulmonary exercise testing in the assessment of an athlete with suspected overlap between physiological adaptation and cardiac pathology. Each domain answers a distinct question and has a characteristic blind spot, indicated in italics; diagnostic confidence derives from their convergence rather than from any single modality. As with Figure 2, the arrangement reflects the authors’ synthesis rather than a validated pathway. CMR, cardiovascular magnetic resonance; ECV, extracellular volume; SCD, sudden cardiac death; BP, blood pressure; VE/VCO_2_, ventilatory equivalent for carbon dioxide.

During the preparation of this manuscript, the authors used Claude (Anthropic; Claude Sonnet 5 and Claude Opus 4.8 models), a large language model-based AI assistant, at several stages of the writing process: (i) to retrieve and screen candidate references identified through the literature search described in Section 2.1; (ii) to draft and structure the narrative text of each section based on direction, clinical framing, and interpretive judgement provided by the authors; (iii) to generate the schematic diagrams shown in Figure 1, Figure 2 and Figure 3, using data-visualization code written according to content and design specifications provided by the authors; and (iv) to format the manuscript according to the journal template. All AI-assisted output, including every citation, reported data point, and figure, was independently verified by the authors against the original primary sources, fact-checked, and edited for scientific accuracy and clarity. The authors reviewed and take full responsibility for the accuracy, integrity, and originality of the final content of this publication, consistent with MDPI’s policy on the use of generative AI in scholarly writing.

## 3. Fundamentals of Cardiopulmonary Exercise Testing in Athletes

### 3.1. Testing Protocols and Core Parameters

CPET couples a symptom-limited, graded exercise protocol, performed on a treadmill or a cycle ergometer using either a ramp or an incremental step design, with breath-by-breath analysis of oxygen uptake (VO_2_), carbon dioxide output (VCO_2_), and minute ventilation (VE), together with continuous 12-lead ECG monitoring and intermittent blood pressure measurement [9]. From these raw signals, a standard set of derived parameters characterizes the cardiopulmonary response to exercise. These include peak oxygen uptake (VO_2_peak, confirmed as a true maximal effort by criteria such as a respiratory exchange ratio ≥ 1.10, a heart-rate plateau, or volitional exhaustion); the first and second ventilatory thresholds (commonly termed the anaerobic threshold and the respiratory compensation point); the slope of the relationship between minute ventilation and carbon dioxide production (the VE/VCO_2_ slope, a marker of ventilatory efficiency); oxygen pulse (VO_2_/heart rate, a surrogate of stroke volume); the kinetics of oxygen uptake at the onset of and recovery from exercise; and the chronotropic, blood pressure, and arrhythmic responses to progressive effort [9,10]. Protocols originally designed for sedentary or clinical populations frequently under-challenge highly trained athletes within a clinically useful test duration, so testing laboratories increasingly tailor the ramp rate and total protocol duration to the athlete’s estimated fitness level in order to obtain a genuine maximal effort [9].

### 3.2. Physiological Basis of High Exercise Capacity in Trained Athletes

According to the Fick principle, VO_2_max is the product of maximal cardiac output and the maximal arteriovenous oxygen difference. Chronic high-volume training increases both terms of this equation. Centrally, it produces eccentric left ventricular remodelling, increased end-diastolic volume, and a correspondingly larger stroke volume and maximal cardiac output; peripherally, it increases capillary density, mitochondrial volume, and oxidative enzyme activity in trained skeletal muscle [1,9]. As a result, healthy, well-trained athletes routinely achieve VO_2_peak values that are supra-normal relative to the general population, and that generic predictive equations derived from sedentary or clinical cohorts may even flag as abnormal in the opposite direction [11]. This systematic mismatch between athlete physiology and general-population reference equations is a central reason why CPET interpretation in this group requires athlete-specific, rather than generic, normative data.

### 3.3. Reference Values: The Influence of Sport Discipline, Sex, and Age

Dedicated athlete-derived reference datasets have only recently become available. The Cardiopulmonary Health and Endurance Exercise Registry (CHEER) demonstrated that VO_2_peak prediction equations derived from general-population cohorts were systematically mis-calibrated when applied to endurance athletes, and it proposed athlete-specific equations with improved accuracy [11]. In a cohort of 1033 elite athletes evaluated before the 2023 European Games and the 2024 Paris Olympic Games, Squeo et al. showed that both VO_2_max and oxygen pulse increased progressively across the four ESC 2020 sport-classification categories, from skill disciplines (VO_2_max 36.3 ± 7.9 mL/kg/min; O_2_ pulse 14.9 ± 3.8 mL/beat) through power and mixed disciplines to endurance disciplines (VO_2_max 52.4 ± 9.7 mL/kg/min; O_2_ pulse 22.7 ± 5.8 mL/beat), with meaningful overlap between adjacent categories. This indicates that the ESC morphological classification only partially tracks the underlying functional capacity of the athlete [1,12]. Age, sex, and competitive level introduce further, largely independent variation. At any given discipline and training level, male athletes generally attain higher absolute and body-mass-indexed VO_2_max than female athletes, and cardiorespiratory fitness in both sexes tends to decline gradually with age even among lifelong endurance-trained individuals, a pattern documented across pediatric, adolescent, and adult elite cohorts [13]. Collectively, these findings indicate that a single, generic normative threshold cannot be applied uniformly across the sporting population, and that the clinical interpretation of any individual CPET parameter should, wherever possible, account for discipline, sex, age, and competitive level, a point that recurs throughout the disease-specific sections below.

### 3.4. Practical Limitations of Cardiopulmonary Exercise Testing

Before considering what CPET contributes to specific diagnoses, it is worth being realistic about what constrains its use. The test is resource-intensive. It requires a calibrated metabolic cart, staff trained in both the acquisition and the interpretation of gas-exchange data, and, in the athletic population, physician supervision for the exercise ECG and blood-pressure response. A complete study occupies roughly an hour of laboratory time once preparation and recovery are included. These requirements confine CPET largely to tertiary and specialist sports cardiology centres. In much of the world it is simply not available to the clinician performing the initial assessment, and where it is available, capacity rarely permits its use as anything other than a second-line investigation directed at a specific question. Nothing in this review should be read as proposing CPET as a routine screening tool; it is not, and the volume of athletes screened annually makes it implausible that it could become one.

The quality of the data is also more operator-dependent than the precision of the reported numbers suggests. Calibration drift, leaks around the mouthpiece or face mask, and inconsistent verbal encouragement all affect the values obtained, and the willingness of an athlete to push to genuine exhaustion varies with familiarity, motivation, and the point in the competitive season at which the test falls. Because peak oxygen uptake is only interpretable if a maximal effort was achieved, a submaximal study yields a number that looks quantitative but means little [9,10]. Determination of the ventilatory thresholds introduces a further layer of subjectivity, with meaningful inter-observer variability even among experienced readers, which is precisely what has motivated interest in automated detection [14]. A more specific problem in athletes is the mismatch between the ergometer and the sport: rowers, swimmers, and cross-country skiers frequently reach lower values on a treadmill or cycle than sport-specific ergometry would elicit, and sport-specific equipment is not widely accessible. Variability in acquisition protocols between laboratories compounds all of this and is discussed in Section 8 [15].

One conceptual limitation deserves separate mention, since it is easily lost among the technical ones. CPET measures the functional consequence of disease, not its presence. A cardiomyopathy that has not yet impaired exercise physiology will produce a normal test, and this is a realistic scenario in exactly the early-stage cases where diagnostic help is most wanted. A normal CPET therefore lowers the probability of clinically significant disease but does not exclude it, and it cannot substitute for structural imaging, electrocardiography, or family assessment [1]. Throughout this review, CPET is discussed as a complement to those investigations rather than as an alternative to them.

## 4. Cardiopulmonary Exercise Testing in the Differential Diagnosis of Specific Conditions

### 4.1. Hypertrophic Cardiomyopathy

Hypertrophic cardiomyopathy (HCM) is among the leading causes of exercise-related sudden cardiac death in young competitive athletes, and mild or borderline left ventricular hypertrophy remains one of the most frequent reasons for CPET referral in sports cardiology [16,17]. Peak oxygen uptake alone, however, has limited discriminative value. When athletes with HCM are matched for training status and habitual exercise volume to healthy athletic controls, both groups can display peak VO_2_ values around 25% above the values expected for age and sex, because long-term training itself raises peak VO_2_ in individuals with HCM just as it does in healthy athletes [16,18]. A pooled meta-analysis across predominantly non-athletic HCM populations has confirmed that peak VO_2_ is, on average, reduced compared with healthy sedentary controls, but this general-population signal does not reliably transfer to the specific, higher-fitness subgroup of athletes with HCM, in whom the parameter can be reassuringly, and misleadingly, normal or supra-normal [17,19].

Other CPET-derived parameters appear more informative in this comparison, although the evidence behind them is of uneven directness. The most directly applicable data come from a cohort in which athletes were matched for fitness against individuals with HCM: here, ventilatory efficiency (the VE/VCO_2_ slope) was significantly worse in the HCM group despite similar peak VO_2_ and similar oxygen uptake at the ventilatory threshold, suggesting that impaired gas exchange, rather than aerobic capacity per se, may better separate the two groups [16]. Evidence for oxygen-pulse behaviour is less direct. Its temporal profile during exercise correlates with reduced functional capacity in HCM independently of left ventricular outflow tract obstruction, but this was established in a referral population of patients with HCM rather than in athletes; the comparison with trained individuals rests on the separate observation that flattened or biphasic responses are uncommon in the latter [20]. The same caveat applies to the secondary analysis of the EXPLORER-HCM randomized trial, which showed that pharmacological reduction in outflow tract obstruction with mavacamten improved several CPET metrics beyond peak VO_2_ [21]. That trial enrolled symptomatic patients with obstructive HCM, not athletes. It therefore supports the physiological relevance of these variables without speaking directly to their diagnostic performance in a trained population. Exercise-induced ventricular arrhythmia and an attenuated or hypotensive blood-pressure response to exercise, although not unique to CPET, remain important components of the same test and continue to inform risk stratification and eligibility discussions in athletes with confirmed or suspected HCM [17].

### 4.2. Dilated Cardiomyopathy and the Left Ventricular “Grey Zone”

Vigorous endurance training can produce marked left ventricular cavity enlargement and a mildly reduced resting ejection fraction that overlaps, on morphological grounds alone, with early or mild dilated cardiomyopathy (DCM), a distinction commonly referred to as the left ventricular “grey zone” [7,8]. The evidence here is more directly applicable than in HCM, because the key studies recruited athletes themselves rather than inferring backwards from clinic populations. In a comparative study of male athletes with LV dilatation and ejection fraction below 55%, control athletes, and patients with DCM, exercise stress echocardiography proved the single most discriminating investigation. Ninety-six percent of grey-zone athletes increased their LV ejection fraction by more than 11 percentage points from rest to peak exercise, compared with only 23% of patients with DCM, and 92% of grey-zone athletes reached a peak ejection fraction above 63%, compared with 17% of DCM patients; failure to meet either cut-off carried a sensitivity of 77–83% and a specificity of 92–96% for DCM [7]. These are wide separations, but the conditions under which they were obtained deserve emphasis. The study was conducted at a single specialist centre, included only male athletes, and compared groups whose diagnostic labels had already been assigned. Diagnostic accuracy estimated in that setting is generally more favourable than what the same cut-offs achieve in an unselected screening population, and neither threshold has yet been reproduced by an independent group. With that caveat, the findings support the principle that preserved or augmented contractile reserve under exercise stress, rather than resting morphology, is the functional signature of physiological remodelling in this context.

This distinction is not absolute. In older, veteran endurance athletes, a population increasingly seen in sports cardiology clinics, the overlap with mild DCM can be closer, and some patients with confirmed DCM are able to meaningfully increase VO_2_max and ejection fraction during exercise, which narrows the discriminative margin of exercise-based testing [8]. In this population, cardiovascular magnetic resonance-based tissue characterization adds complementary value: septal fibrosis was essentially absent in veteran athletes with LV dilatation but present in a substantial minority of patients with mild DCM, and native T1 and extracellular volume values differentiated the two groups with good accuracy (area under the curve 0.85–0.89) [8]. Advanced echocardiographic deformation imaging, including global longitudinal strain and atrial reservoir strain, has also been proposed as a complementary tool, since it can detect subtle contractile impairment before the ejection fraction declines, although reference ranges specific to trained athletes are still being defined [5]. A further, long-established discriminator is the response to a defined period of detraining. Because physiological adaptation is reversible, whereas, pathological remodelling is not, regression of chamber dilatation and normalization of function after several weeks of reduced training support a benign diagnosis, and this maneuver remains a useful adjunct when non-invasive testing is equivocal [1]. Taken as a whole, the current evidence supports a combined, cascade-based strategy for the DCM “grey zone,” using exercise echocardiography or CPET-integrated stress imaging as a first-line functional test and escalating to CMR tissue characterization or a detraining period when the functional response remains equivocal, rather than relying on any single exercise parameter in isolation.

### 4.3. Arrhythmogenic Cardiomyopathy

Arrhythmogenic cardiomyopathy (ACM) is a heritable, progressive fibro-fatty myocardial disease that is disproportionately triggered by intense exercise and remains a leading cause of sudden cardiac death in young athletes, which makes its distinction from physiological right ventricular adaptation to training a particularly high-stakes diagnostic task [22]. Unlike in HCM, where aerobic capacity itself can be misleadingly preserved, exercise tolerance in ACM is typically at or below the value predicted for age and sex, in contrast to the above-predicted exercise tolerance generally seen in healthy athletes; this makes overall exercise capacity a somewhat more useful, if still imperfect, discriminator in this specific condition [22]. The arrhythmic response to exercise is particularly informative. An increase in the frequency or complexity of premature ventricular beats during exercise testing is uncommon in healthy athletes and, in the appropriate clinical context, raises concern for an underlying cardiomyopathy; whereas, a reduction in ectopic burden with increasing exercise intensity is reassuring of a benign, physiological origin [22]. Exercise-induced ventricular arrhythmias have been reported in around 40% of patients with confirmed ACM, particularly in those with more extensive biventricular structural involvement [22]. That figure comes from disease cohorts assembled in specialist centres, not from athletes under evaluation, so it describes what ACM looks like once diagnosed rather than the yield of the test in a screening setting. It supports using the exercise arrhythmic response as one element of a broader work-up rather than as a stand-alone criterion.

As with dilated cardiomyopathy, functional exercise testing performs best when integrated with structural imaging rather than used in isolation. Cardiovascular magnetic resonance comparisons between confirmed ACM and healthy athletes have found that a meaningful proportion of ACM patients do not fulfil formal imaging Task Force Criteria, while some abnormal structural findings can be identified outside these criteria. This reinforces that no single imaging or functional threshold is sufficient, and that a systematic, multiparametric approach, combining family and personal history, resting and exercise ECG, structural and tissue-characterizing imaging, and, where indicated, genetic testing, offers the most reliable pathway to distinguishing ACM from athletic remodelling [22,23].

### 4.4. Thoracic Wall Deformities: Pectus Excavatum as a Confounder

A less widely recognized but clinically important confounder in this differential-diagnosis landscape is pectus excavatum, the most common congenital chest wall deformity. It can independently produce exertional dyspnoea, reduced exercise capacity, and electrocardiographic changes, including right bundle branch block, T-wave inversion, and QS complexes, that mimic several of the conditions discussed above, including arrhythmogenic right ventricular cardiomyopathy and prior myocardial infarction [6]. In athletes with pectus excavatum, sternal depression and cardiac compression can impair right ventricular diastolic filling and stroke-volume augmentation during exercise, producing a pattern of reduced exercise capacity that is mechanical and cardiovascular in origin rather than reflective of an inherited cardiomyopathy [6]. Here, CPET plays a distinct and complementary role. Measures of inspiratory capacity and tidal volume can identify dynamic hyperinflation or ventilatory limitation as the dominant mechanism of exertional symptoms, while oxygen pulse, relatively independent of training status in this population, has been proposed as a more specific marker of the degree of diastolic restriction than peak VO_2_ alone, which in trained athletes with pectus excavatum can remain within a deceptively normal range [6]. Recognizing this entity, and using CPET to separate mechanical from cardiovascular limitation, helps avoid both unnecessary disqualification and the false reassurance of a normal resting echocardiogram in athletes whose chest-wall anatomy alone can explain their symptoms and ECG findings.

Across all four conditions reviewed above, no single CPET parameter reliably separates physiological adaptation from pathology in isolation. Peak VO_2_ is particularly unreliable in HCM, where training status can normalize or even supra-normalize this value; ventilatory efficiency, oxygen-pulse kinetics, and the exercise arrhythmic response add discriminative information across several conditions, but each carries its own false-positive and false-negative profile. The strength of the underlying evidence also differs markedly between these conditions, and this asymmetry deserves to be stated plainly. Only in the dilated cardiomyopathy “grey zone” have discriminatory thresholds been derived from athletes evaluated as such [7,8]. Much of what is said about oxygen-pulse kinetics, ventilatory efficiency, and arrhythmic burden in the other conditions comes from patients recruited in cardiomyopathy clinics, where age, symptom status, and habitual activity differ substantially from those of a competing athlete. Applying such findings to athletes is reasonable and is what current practice does, but it remains an extrapolation, and the resulting recommendations should be read as provisional. Table 1 indicates, for each condition, whether the listed patterns rest on athlete-derived or indirect evidence. It is equally worth stating what the literature does not establish. The studies reviewed here were not designed to compare CPET variables against one another, and we identified no head-to-head analysis showing that any single parameter outperforms the others, nor prospective work defining how they should be weighted when they point in different directions. Sample sizes are mostly modest, recruitment is typically from tertiary referral centres, and follow-up to hard clinical endpoints is largely absent. Any ranking of these measurements, including the emphasis placed on them in this review, therefore rests on indirect comparison across studies of differing design and populations rather than on direct evidence. The consistent lesson from the literature reviewed in this section is that CPET is most valuable as one component of a systematic, multiparametric evaluation, alongside family and personal history, resting and exercise ECG, and structural or tissue-characterizing imaging, rather than as a stand-alone arbiter of sports eligibility.

### 4.5. Prognostic Value and the Prediction of Adverse Outcomes

Everything discussed so far concerns diagnosis. A separate question, and one clinicians ask frequently, is whether CPET can indicate which athletes will go on to suffer an adverse event. The prognostic literature is strongest in hypertrophic cardiomyopathy. A meta-analysis of sixty studies comprising almost thirteen thousand patients examined CPET both for characterization and for prognostication, with mortality assessed through pooled hazard ratios, and found reduced peak oxygen uptake to be associated with adverse outcome [19]. A separate systematic review reached a consistent conclusion, reporting that lower VO_2_max is seen consistently in patients with HCM at higher cardiovascular risk [17]. Impaired ventilatory efficiency and an abnormal blood-pressure response during exercise carry similar associations, which is why the latter has long featured in risk assessment for this condition [17]. Beyond HCM, the American Heart Association statement on congenital heart disease concluded that CPET variables carry strong prognostic value, particularly when tests are repeated over time [24], and in arrhythmogenic cardiomyopathy the burden of exercise-induced ventricular arrhythmia relates to the extent of structural involvement and to arrhythmic risk [22].

These findings are genuine, but transferring them to the athlete in front of us requires two steps that the literature does not support. The first concerns the population. Prognostic models in HCM and congenital heart disease were built in diagnosed patients under clinical follow-up, most of them not athletes, and many of them symptomatic. Predicting death or heart-failure progression in that group is a different task from predicting sudden death during competition in a young person whose diagnosis is uncertain. The second concerns the outcome itself. Most of these analyses pooled endpoints such as all-cause mortality, transplantation, and hospitalization for heart failure. Sudden cardiac death during sport is a small and mechanistically distinct component of that composite, and a variable that predicts the whole does not necessarily predict the part.

There is also a statistical obstacle that no improvement in test performance can overcome. Sudden cardiac death in athletes is rare, with reported incidence on the order of a few events per million person-years and a roughly ten-fold difference between male and female athletes [25]. When the event rate is that low, even a test with excellent sensitivity and specificity yields a positive predictive value close to zero, and the overwhelming majority of abnormal results in an unselected athletic population will be false alarms. This is not an argument against measuring these variables. It is an argument about how their results should be described. CPET cannot function as a stand-alone predictor of sudden death in athletes, and we would avoid presenting it to an athlete in those terms.

Where CPET does contribute is narrower but still useful. In an athlete with an established diagnosis, serial testing describes a functional trajectory, and deterioration in peak VO_2_, ventilatory efficiency, or the arrhythmic response over successive assessments is more informative than any single value and may reasonably prompt reassessment of eligibility [24]. Reduced functional capacity also identifies athletes in whom the disease is no longer physiologically silent, which is relevant context for a shared decision about continued competition [1,3]. What is missing, and what would change this picture, is prospective follow-up of athletes with confirmed cardiovascular conditions who continue to train under surveillance, with CPET performed serially and hard clinical endpoints recorded. We return to this gap in Section 8.

## 5. Special Populations

### 5.1. Veteran (Master) Athletes

Veteran, or “master,” athletes, typically defined as competitive or highly active individuals aged 35 years or older, represent a rapidly growing population in sports cardiology clinics and pose a distinct set of diagnostic challenges from those discussed in Section 4. In young athletes, sudden cardiac death is predominantly attributable to inherited cardiomyopathies and channelopathies. In master athletes, by contrast, the dominant cardiovascular risk is acquired coronary artery disease, alongside a higher prevalence of atrial arrhythmias, aortic dilatation, and myocardial fibrosis than in age-matched sedentary peers [26]. A recent joint clinical consensus statement from the European Association of Preventive Cardiology and the American College of Cardiology emphasized that existing management guidelines for these conditions are derived almost exclusively from sedentary, symptomatic patients, which limits their direct applicability to highly trained older athletes, and it called for an individualized, shared decision-making approach that weighs cardiovascular risk against the athlete’s performance and health goals [26].

Exercise testing, whether as a standard exercise ECG or a full CPET, remains central to this individualized assessment, because it can provoke silent myocardial ischaemia and exercise-induced arrhythmias that are not apparent at rest, and it defines objective exercise capacity relevant to eligibility discussions [26]. Its performance is imperfect, however. In a study of 506 consecutive master athletes undergoing pre-participation screening, none of the three contemporary ECG interpretation criteria (2017 International, 2010 ESC, and 2013 Seattle) achieved high diagnostic accuracy for conditions associated with sudden cardiac death; the Seattle criteria performed best (area under the curve 0.81) and the International criteria worst (0.73) [27]. These findings reinforce that exercise-based testing in veteran athletes should be interpreted as part of a multimodal screening pathway, combining history, resting and exercise ECG, and, in doubtful cases, advanced imaging such as cardiac magnetic resonance or coronary computed tomography angiography, rather than as a stand-alone gatekeeper for continued sports participation [26,27].

### 5.2. Women Athletes

Women remain markedly underrepresented both in the athlete populations most frequently studied and in the reference datasets used to interpret their results. Sports-related sudden cardiac death is approximately ten-fold less common in female than in male athletes, at an estimated incidence of around 0.19 versus 2.63 per million person-years, respectively, a disparity that persists even after accounting for similar training exposure [25]. While reassuring, this large sex difference in absolute event rates has also meant that female athletes are underpowered in most single-centre and even multicentre cohorts, and that pre-participation screening criteria, imaging thresholds, and, by extension, CPET reference equations have historically been derived from predominantly male populations [25,28]. The CHEER endurance-athlete reference dataset discussed in Section 3, for example, comprised only 31% female participants [11], and elite-athlete CPET studies across age, sex, and discipline continue to report systematically lower absolute and body-mass-indexed VO_2_max in women than in men within the same sport and competitive tier. This difference is attributable in part to differences in hemoglobin mass, cardiac chamber dimensions, and body composition rather than to any difference in training adaptation [13].

These physiological differences mean that applying male-derived CPET thresholds to female athletes risks both under- and over-interpretation of borderline findings, and the evidence base explicitly linking sex-specific CPET parameters to the differential diagnosis of the cardiomyopathies discussed in Section 4 remains sparse. Expanding female representation in athlete exercise-testing registries, and validating sex-specific reference equations and disease-specific CPET cut-offs, is among the clearest and most actionable evidence gaps identified in this review [25,28].

### 5.3. Pediatric Athletes and Congenital Heart Disease

Athletes with congenital heart disease (CHD) represent a third population in which CPET interpretation departs substantially from the adult, structurally normal athlete framework described in Section 3 and Section 4. Reflecting this, recent sports-eligibility guidance has explicitly moved away from blanket activity restrictions for athletes with CHD in favour of an individualized approach centred on longitudinal surveillance and, specifically, cardiopulmonary exercise testing [3]. A dedicated 2025 scientific statement from the American Heart Association on CPET interpretation across the lifespan in CHD reinforced this shift, noting that even patients with simple lesions such as atrial or ventricular septal defects frequently show suboptimal cardiorespiratory fitness relative to unaffected peers, that CPET variables carry strong prognostic value, particularly on serial testing, and that CPET is especially informative in more complex physiologies such as the Fontan circulation, Ebstein anomaly, and repaired tetralogy of Fallot [24].

Interpretation in this population poses additional, largely pediatric-specific challenges. Exercise protocols must be adapted to the child’s or adolescent’s developmental stage rather than to a fixed adult template, and reference values must account for the substantial, ongoing changes in body size and cardiopulmonary maturation that occur throughout childhood and adolescence. This is a further illustration of the general principle, introduced in Section 3, that no single normative dataset can be applied uniformly across the sporting population [24]. As with veteran and female athletes, prospective, longitudinal CPET data specific to young athletes with CHD remain comparatively limited and represent a priority area for future research.

## 6. Emerging Technologies: Artificial Intelligence in CPET Interpretation

Manual identification of ventilatory thresholds and other qualitative CPET features remains subject to meaningful inter-observer variability, which has motivated growing interest in artificial intelligence (AI) as a tool to standardize and accelerate CPET interpretation [14]. Deep learning models trained on large repositories of CPET recordings, such as Oxynet, have been developed to automatically identify the first and second ventilatory thresholds from breath-by-breath gas exchange and ventilatory data; in validation against experienced human evaluators, such algorithms have shown accuracy and reliability comparable to expert visual inspection, with mean between-method biases on the order of tens of millilitres per minute [14]. Beyond threshold detection, large language models have also been explored for higher-level interpretation of complete CPET reports. In one cross-sectional study, GPT-3.5 and GPT-4 identified normal CPET results with high specificity, and their combined output achieved 91% sensitivity and 98% specificity for ruling out an abnormal test, although accuracy for correctly classifying the type of abnormality (cardiovascular versus respiratory limitation) was lower, and was specifically degraded at low work rates and peak oxygen consumption [29].

These results are encouraging, but the population in which they were obtained matters more here than it usually would. Both the threshold-detection algorithms and the language-model work were developed and validated on sedentary or clinical cohorts. To our knowledge, none has been trained on athletes and none has been tested in them. We would argue that this is not simply a gap in external validity to be closed later, because it bears directly on the problem this review addresses. An algorithm learns the relationship between gas-exchange patterns and diagnostic labels from the examples it is shown. If trained athletes are absent from those examples, there is little reason to expect it to have learned where supra-normal physiology ends and early disease begins, which is precisely the boundary a sports cardiologist needs help with. The finding that low, rather than high, exercise capacity predicted inaccurate interpretation in the language-model study [29] shows that performance does vary with position on the physiological spectrum, although it describes the opposite end from the one at issue here. AI-based interpretive tools could therefore reproduce, rather than resolve, the same reference-population mismatch already described for conventional predictive equations, unless they are specifically trained and validated on athlete-derived data. Broader applications of AI in sports cardiology, including automated ECG interpretation, imaging-based phenotyping, and integration of wearable-device data, are similarly promising but remain at an early stage, and none has yet been formally incorporated into clinical eligibility guidelines [30,31].

It follows that we would place AI-assisted CPET interpretation in the category of promising research rather than of clinical tools ready for use, and the distinction seems worth stating plainly. No such system has been validated in an athletic population, none is incorporated into any pre-participation pathway or eligibility recommendation, and none has been assessed against clinical outcomes in this setting [30,31]. There is also a practical hazard familiar from other areas of diagnostic automation. An algorithm that produces a confident-looking output can anchor the reader’s judgement even when it is wrong, and that risk falls hardest on less experienced interpreters, who are also the readers most likely to find automated assistance appealing. Until athlete-specific training data and prospective validation exist, our view is that these tools should inform research questions rather than decisions about whether an individual athlete competes.

## 7. A Practical Diagnostic Framework

The evidence reviewed in the preceding sections can be synthesized into a practical, stepwise framework for integrating CPET into the assessment of an athlete with a suspected overlap between physiological adaptation and cardiac pathology, consistent with the individualized, shared decision-making approach advocated by current sports cardiology guidelines [1,3]. We want to be clear about the status of what follows. The sequence set out below, and in Figure 2, is our own reading of the studies discussed in Section 3, Section 4, Section 5 and Section 6. It is not an externally validated diagnostic pathway, it has not been tested prospectively against clinical outcomes, and no professional society has endorsed it. Individual components draw on guideline recommendations, particularly the screening step and the emphasis on shared decision-making [1,2,3,4], but the order in which the investigations are arranged and the weight given to each CPET variable reflect our judgement rather than established consensus. Readers should treat it as a starting point for structuring the work-up, to be adapted to local resources and to the individual athlete.

### 7.1. A Stepwise Approach

First, every athlete undergoes standard pre-participation screening, comprising a structured history, physical examination, and resting 12-lead ECG interpreted according to international athlete-specific criteria [2,4]. Findings are triaged into normal, borderline, or abnormal categories. Isolated normal or borderline findings in an asymptomatic athlete with no relevant family history generally do not require further testing; whereas, abnormal findings, multiple borderline findings, or symptoms such as exertional chest pain, dyspnoea disproportionate to training load, palpitations, or syncope prompt escalation [2,4].

Second, when escalation is warranted, CPET is performed as a first-line functional test alongside, or ahead of, structural imaging. Beyond peak oxygen uptake, which as discussed in Section 4 is frequently misleading in this population, the variables that in our reading carry the most information are ventilatory efficiency (the VE/VCO_2_ slope), the absolute value and temporal behaviour of oxygen pulse, the exercise blood-pressure response, and the frequency and complexity of exercise-induced ectopy. We would stress that this emphasis reflects our synthesis of the studies discussed above and not a validated hierarchy; no trial has established that these variables should take precedence over others in routine practice. Wherever possible these are interpreted against athlete-specific and, ideally, discipline-, sex-, and age-matched reference values rather than generic predictive equations [11,12,13]. Table 1 summarizes the CPET patterns that are generally reassuring versus those that constitute red flags for the four conditions discussed in Section 4, and Figure 2 sets out the overall stepwise framework.

Third, CPET findings are integrated with structural and, when indicated, tissue-characterizing imaging rather than interpreted in isolation. As detailed in Section 4, exercise stress echocardiography and cardiac magnetic resonance add complementary discriminative value in the DCM “grey zone” and in suspected arrhythmogenic cardiomyopathy, and they are essential to identify structural confounders such as pectus excavatum. Family history and, where appropriate, genetic testing complete the diagnostic work-up in athletes whose phenotype remains ambiguous after functional and imaging assessment [6,7,8,22,23]. Figure 3 sets out how these modalities relate to one another. Each answers a different question, and each carries a characteristic blind spot, which is the reason none of them is dispensable and none is sufficient alone.

Finally, when a truly borderline picture persists despite this multiparametric assessment, management should default to shared decision-making between the athlete, the treating cardiologist, and, where relevant, the athlete’s family and sporting organization, weighing individualized cardiovascular risk against the athlete’s competitive goals rather than defaulting uniformly to a restrictive or permissive position [1,3]. This framework does not replace clinical judgement or the disease-specific nuance discussed throughout this review. It offers a structured starting point that keeps three recurring lessons of Section 3, Section 4, Section 5 and Section 6 anchored in everyday practice: the unreliability of peak VO_2_ in isolation, the need for athlete-specific reference values, and the primacy of a multiparametric approach. Whether following this sequence improves diagnostic accuracy or clinical outcomes compared with current practice is an open question, and one we would encourage others to test.

### 7.2. When CPET Variables and Imaging Disagree

The framework above describes a tidy situation in which findings point the same way. Clinical practice is rarely that obliging. The athlete who arrives with a uniformly abnormal study is comparatively easy; far more often we are asked to interpret a preserved peak VO_2_ alongside a mildly elevated VE/VCO_2_ slope, an oxygen-pulse curve that flattens late but not convincingly, a handful of exercise-induced ventricular beats, or a wall thickness of 13 mm with everything else unremarkable. Because this is where the diagnostic difficulty actually lies, it is worth setting out how we approach it, while acknowledging that this section rests on clinical reasoning and the physiological principles discussed earlier rather than on evidence from studies designed to answer the question.

Our first question is whether an isolated abnormality is real. Single CPET variables are more fragile than they appear: ventilatory efficiency is sensitive to hyperventilation, anxiety, and breathing pattern during the test, and an elevated VE/VCO_2_ slope in an athlete who is unfamiliar with the mouthpiece is more often an artefact than a sign of disease [9,10]. Repeating the test, ideally after the athlete has become accustomed to the equipment, resolves a proportion of these cases without any further investigation. The same applies to oxygen pulse, whose temporal profile is difficult to read reliably when the ramp is too steep for the individual’s fitness and the test ends after only a few minutes [9]. Before escalating, we would want to be satisfied that the protocol was appropriate for the athlete and that a genuine maximal effort was reached.

When an abnormality is reproducible, its weight depends heavily on what surrounds it. A borderline CPET finding in an athlete with no symptoms, an unremarkable family history, and a normal athlete-criteria ECG carries a very different implication from the same finding in someone with exertional syncope or a first-degree relative who died suddenly. In the first situation, the pre-test probability of disease is low enough that an isolated functional abnormality seldom changes management on its own, and the reasonable response is structured surveillance rather than restriction. In the second, the same result may justify proceeding to cardiac magnetic resonance or genetic testing even if the remaining CPET variables are normal. Discordance between CPET and imaging is handled the same way: neither modality overrides the other, and a borderline wall thickness or chamber dimension with entirely normal functional reserve is, in our practice, more reassuring than a normal-looking ventricle with impaired contractile reserve on exercise echocardiography [7,8].

Two further considerations help when the picture stays ambiguous. The first is time. Serial testing at intervals of six to twelve months converts a static, uncertain snapshot into a trajectory, and a stable finding over two or three assessments is considerably more reassuring than a single normal study; a supervised period of reduced training can be equally informative where physiology and pathology remain difficult to separate [1]. The second is the specific pattern in front of us, since not all discordance carries equal concern. Isolated exercise-induced ventricular ectopy that increases in frequency or complexity with workload deserves more attention than an isolated ventilatory abnormality, because the arrhythmic response is the variable most directly linked to the mechanism of sudden death in arrhythmogenic conditions [22]. Conversely, a ventilatory limitation pattern with preserved oxygen pulse should prompt a search for respiratory or chest-wall explanations before the heart is implicated at all [6]. What we would caution against, in every one of these scenarios, is disqualifying an athlete on the strength of one discordant variable. The consequences of that decision are substantial, the diagnostic performance of any single measurement is modest, and the appropriate response to genuine uncertainty is to say so, to keep the athlete under review, and to share the reasoning with them [1,3].

## 8. Limitations and Future Research Directions

This review has several limitations inherent to its narrative design. Literature was not identified through a systematic, pre-registered search protocol, and the search was restricted to English-language publications, which may have introduced selection bias and could have omitted relevant regional data, particularly given the substantial geographic and ethnic heterogeneity discussed below. Sports cardiology is also a rapidly evolving field, and several of the studies cited here were published within the past twelve months; the specific quantitative thresholds discussed should therefore be expected to be refined as larger, prospective datasets become available.

A more fundamental limitation concerns the state of the underlying evidence base itself, rather than this review’s synthesis of it. A recent survey of CPET laboratories caring for athletes and cardiopulmonary patients found substantial heterogeneity in testing protocols, supervision requirements, and interpretation standards across centres, with no consensus on which specific parameters should be routinely reported or how borderline results should be escalated [15]. This heterogeneity complicates direct comparison of findings across the studies reviewed in Section 4 and Section 5 and represents a practical barrier to translating the available evidence into consistent clinical practice. On reflection, we suspect this problem deserves more weight than it usually receives, and that it may limit implementation as much as the missing athlete-specific data discussed above. The reason is that CPET reference values are not properties of the athlete alone; they are properties of the athlete and the protocol together. Peak oxygen uptake measured on a treadmill is consistently higher than the value obtained from the same individual on a cycle ergometer, and the steepness of the ramp influences both peak VO_2_ and the work rate at which the ventilatory thresholds appear [9,10]. Ventilatory efficiency is equally method-dependent, since the VE/VCO_2_ relationship can be reported as a slope calculated across the whole test or only to the ventilatory threshold, and as a ratio at a defined point, with values that are not interchangeable. Threshold determination itself varies between the V-slope method, ventilatory equivalents, and combined approaches, and increasingly between manual and automated identification [14].

The practical consequence is that an athlete-specific reference equation cannot simply be lifted from the registry that produced it and applied in another laboratory. The CHEER equations, for instance, were derived under particular acquisition conditions [11], and a centre using a different ergometer, ramp protocol, or threshold-detection method may not be measuring quite the same quantity. This matters most for exactly the borderline cases described in Section 7.2, where the difference between a normal and an abnormal classification may be smaller than the variation introduced by methodology. Producing better normative data will therefore accomplish less than it should unless it is accompanied by agreement on how the test is performed and reported. In our view the minimum requirements would be a defined ergometer and ramp strategy for athletic testing, explicit criteria for accepting a test as maximal, a stated method for threshold determination, and a reporting set that includes ventilatory efficiency, oxygen pulse, and the arrhythmic and blood-pressure response rather than peak VO_2_ alone. None of this requires new technology, and a consensus document from the relevant societies would be sufficient to achieve it.

As highlighted throughout Section 5, the athlete populations and reference datasets underpinning current CPET interpretation remain skewed toward young, White, male endurance athletes, and this imbalance extends beyond the sex, age, and congenital-disease dimensions already discussed. Athletes’ cardiac adaptation varies systematically by ethnicity and geographic ancestry, including in the prevalence of ECG repolarization patterns and left ventricular wall thickness that overlap with cardiomyopathy. Yet a recent systematic review found that current international ECG criteria carry a more than two-fold higher false-positive rate in Black compared with White athletes, and equivalent, prospectively validated CPET reference data across ethnic groups are, to our knowledge, unavailable [32]. Athletes with disabilities represent another comparatively neglected population. A recent United Kingdom cohort study found that elite para-football players were three times more likely than matched non-para players to be diagnosed with a cardiac condition requiring management, yet the authors specifically noted that the cardiac characteristics of para-athletes remain under-represented in the literature relative to their unique cardiovascular physiology and risk profile [33]. Extending athlete-specific CPET reference values and disease-specific interpretive criteria to these populations is at least as pressing a research priority as the sex- and age-related gaps discussed in Section 5.

A closely related gap concerns the paucity of prospective, longitudinal data on athletes with confirmed cardiovascular conditions who continue training and competing under surveillance. This is precisely the population in whom serial CPET could be most informative for eligibility decisions; yet, current evidence is dominated by cross-sectional comparisons, as illustrated by the DCM and HCM literature discussed in Section 4. Registries that prospectively follow athletes with confirmed cardiomyopathies, arrhythmogenic conditions, or repaired congenital heart disease through serial CPET, alongside imaging and clinical outcomes, would directly address the evidence gap that motivated this Special Issue and would allow eligibility recommendations to move from expert consensus toward outcomes-based evidence.

Finally, as discussed in Section 6, the emerging generation of AI-assisted CPET interpretation tools has not yet been validated in athletic populations. Prospective validation studies designed for this purpose, ideally incorporating the diverse populations discussed above rather than replicating existing gaps, represent a further priority for future research.

## 9. Conclusions

Cardiopulmonary exercise testing offers a complementary, functional perspective to the structural and electrical assessment of athletes in whom physiological cardiac adaptation must be distinguished from early or mild cardiac pathology, the central diagnostic dilemma addressed throughout this Special Issue. Across hypertrophic cardiomyopathy, dilated cardiomyopathy, arrhythmogenic cardiomyopathy, and thoracic wall deformities such as pectus excavatum, the recurring lesson of the evidence reviewed here is that peak oxygen uptake alone is an unreliable arbiter, because it can remain deceptively normal, or even supra-normal, in conditions that carry genuine risk. Ventilatory efficiency, the absolute value and temporal behaviour of oxygen pulse, the exercise blood-pressure and arrhythmic response, and, where available, exercise-based imaging appear to add information beyond peak VO_2_. That impression rests on observational studies of modest size rather than on direct comparison between parameters, so it should be held with corresponding caution. Each measurement carries its own limitations, and none performs reliably as a stand-alone criterion. The stepwise framework set out in Section 7 organizes these observations into a practical sequence, but it is the authors’ synthesis of the literature reviewed here: it has not been tested prospectively against clinical outcomes, no professional society has endorsed it, and it is intended to be adapted to local resources and to the individual athlete rather than applied as a validated algorithm.

Realizing the full diagnostic potential of CPET in sports cardiology will depend on evidence that does not yet fully exist: athlete-specific reference values that account for sport discipline, sex, age, and ethnicity; prospective, longitudinal data on athletes who continue to train and compete under surveillance for confirmed cardiovascular conditions; harmonized testing and interpretation protocols across laboratories, without which better reference data will not transfer into practice; and rigorous, athlete-specific validation of emerging AI-assisted interpretive tools. Until these gaps are closed, CPET is best regarded as one component of a systematic, multiparametric, and individualized evaluation, integrated with clinical history, imaging, and, where appropriate, genetic testing, and placed ultimately in the service of shared decision-making between the athlete and their care team.

## Figures and Tables

**Table 1:** CPET patterns of physiological adaptation versus disease-specific red flags. The final column indicates whether the listed patterns derive from studies performed in athletes or are extrapolated from non-athletic cardiomyopathy populations.

Condition	Reassuring (“Athlete’s Heart”) Pattern	Red Flag Warranting Further Work-Up	Evidence Base
Hypertrophic cardiomyopathy	Normal or supra-normal peak VO_2_; normal VE/VCO_2_ slope; normal, monotonic O_2_-pulse kinetics [16,18,20]	Elevated VE/VCO_2_ slope despite preserved peak VO_2_; flattened or biphasic O_2_-pulse response; complex exercise-induced ventricular ectopy; attenuated/hypotensive blood-pressure response [16,17,20]	Mixed. VE/VCO_2_ athlete-matched [16]; O_2_-pulse and drug-trial data from non-athletic cohorts [20,21]
Dilated cardiomyopathy/LV “grey zone”	LV ejection fraction increase >11 percentage points from rest to peak exercise; peak LVEF > 63% on stress echocardiography [7]	Failure to augment LVEF on exercise; persistently low peak VO_2_ out of proportion to training history; septal fibrosis on CMR [7,8]	Athlete-derived; cut-offs established directly in athletes [7,8]
Arrhythmogenic cardiomyopathy	Exercise tolerance above the value predicted for age/sex; reduction in ectopic burden with increasing exercise intensity [22]	Exercise tolerance at or below predicted; increase in frequency/complexity of ventricular ectopy with exercise, especially with biventricular structural involvement [22,23]	Mixed; largely from ACM referral cohorts [22,23]
Pectus excavatum	Ventilatory/mechanical limitation pattern (abnormal VT/IC ratio, dynamic hyperinflation) with preserved O_2_-pulse [6]	Reduced O_2_-pulse suggesting genuine diastolic filling/stroke-volume restriction, independent of training status [6]	Limited; small series; no validated cut-offs [6]

## Data Availability

No new data were created or analyzed in this study. Data sharing is not applicable to this article.
